# Strategic cortical engagement with basal implants for anterior ridge rehabilitation: A clinical case report

**DOI:** 10.6026/973206300211820

**Published:** 2025-07-31

**Authors:** Muthupriya S, Uma Subbiah, Saranya Mohan, Krishna Kumar, Vijayalakshmi R, Jaideep Mahendra, D Shanmuga Prasanth

**Affiliations:** 1 Department of Periodontics, Meenakshi Ammal Dental College and Hospital, Faculty of dentistry, MAHER, Chennai, Tamil Nadu, India

**Keywords:** Atrophic ridges, osseointegration, basal implants, immediate placement, esthetic zone

## Abstract

Tooth loss and ridge resorption complicate traditional implant therapy, often requiring grafts and prolonged healing for success. Basal implants offer a solution by anchoring into dense cortical bone, enabling immediate loading and superior primary stability. This case involved missing mandibular incisors and an extracted maxillary lateral incisor, rehabilitated using single-piece basal implants without grafting. Synthetic Novabone Putty supported regeneration in deficient areas. With atraumatic extractions, bicortical stabilization, and timely restoration, both function and esthetics were efficiently restored.

## Background:

Tooth loss leads to progressive alveolar atrophy, which makes implant placement especially challenging. Achieving optimal outcomes in the esthetic zone demands both strategic positioning and sufficient bone volume. In scenarios where vertical height is limited and cost-effectiveness is a concern, basal implants offer a reliable alternative. Basal implants, including Basal Osseo Integrated (BOI) and Basal Cortical Screw (BCS) types, are specifically designed to harness the dense basal bone for enhanced stability and infection resistance. These screwable implants facilitate immediate fixation and are often placed without the need for flaps or extensive bone preparation, significantly reducing the invasiveness of the procedure. By bypassing the more vulnerable trabecular alveolar bone, basal implants reduce the incidence of peri-implantitis and eliminate the need for multiple surgeries often required in conventional Implantology [1, 2-3]. Basal implantology, also known as bicortical or cortical implantology was pioneered by Dr. Jean Marc Julliet in 1972 and further refined by Dr. Gerard Scortecci and Dr. Stefan Idhe in 1997. The concept was developed in response to the limitations of traditional two-stage implants that relied on bone augmentation and lengthy healing times. Immediate implant placement, originally proposed by Dr. Wilfried Schulte, remains a viable technique for preserving alveolar bone architecture and reducing treatment duration [4].

An additional approach in ridge management is Guided Bone Regeneration (GBR), which, although effective, is often more time-consuming and technique-sensitive. In comparison, the use of synthetic bone graft materials like NovaBone Putty provides a simpler, yet clinically effective alternative. NovaBone Putty is an alloplastic biocompatible material with proven osteoconductive properties. Composed of calcium phosphate silicate and enriched with polyethylene glycol and glycerol, it offers excellent handling characteristics and enhances the biological environment for bone regeneration [5]. Therefore, this case report demonstrates that the combined use of basal implants and NovaBone Putty offered a swift, minimally invasive, and stable approach for rehabilitating a resorbed anterior ridge with immediate functional and esthetic outcomes.

## Case report:

This case report adheres to the CARE guidelines (UK Equator Centre) as a checklist to ensure comprehensive reporting.

## Patient information:

A 32-year-old male patient presented to the Department of Periodontics with a chief complaint of missing lower anterior teeth for the past five years and supra-eruption of upper anterior teeth. The patient reported no relevant medical history or use of medications.

## Clinical findings:

Intra-oral examination revealed inadequate oral hygiene, reflected by a poor Oral Hygiene Index-Simplified (OHI-S) score. Clinical assessment showed the absence of teeth 31 and 41 (mandibular central incisors), likely due to periodontal disease. Tooth 22 (maxillary left lateral incisor) exhibited supra-eruption, and extensive carious involvement was noted in tooth 24 (maxillary left first premolar). After discussing various treatment options, a cone-beam computed tomography (CBCT) scan was performed to evaluate the available bone levels.

## Therapeutic intervention:

Upon evaluation of CBCT and intra-oral examination, Basal implants were selected which are crucial in the anterior region for esthetics, function and stability. They utilize dense basal bone, ensuring strong anchorage even in resorbed ridges. Due to the observed resorption, NovaBone putty, a bone graft material, was utilized to enhance bone regeneration. It is particularly helpful in cases with compromised bone volume, ensuring better osseointegration and long-term success of basal implants. With patient consent, it was decided to place single-piece basal implants in the 31 and 41 regions and to extract the 22 region tooth, followed by immediate basal implant placement using NovaBone putty as a graft material.

## Surgical intervention:

For aseptic precautions, the patient was asked to rinse mouth with 0.12% chlorhexidine mouth rinse solution for 2 minutes. Local was administered using supraperiosteal infiltration with 2% lignocaine HCl for the extraction of tooth 22. For implant placement in regions 31 and 41, bilateral mental nerve blocks were given. Tooth 22 was atraumatically extracted using maxillary anterior forceps without damaging the surrounding thin alveolar plates of bone and minimally lacerating the soft tissues. Curettage and irrigation of extraction sockets using saline were also performed. Following this immediate implant placement was done in 22 regions. It was initiated with Mesial and distal incisions using no. 15 scalpel and the pyramidal mucoperiosteal flap were elevated using a periosteal elevator. An osteotomy was done using the pilot drill for socket preparation and drilling to a depth of 3mm beyond the socket. The basal implant was placed with hand pressure using a plastic cap and a ratchet wrench. Follow by the placement of Nova bone putty. Tissue was approximated using a simple interrupted suture. In the edentulous region, 31-41 mid-crestal incisions were made using no. 15 scalpel and mucoperiosteal flap was elevated using Molt's no. 9 periosteal elevator. The pilot drill was placed on the ridge. Basal implants were placed, followed by the placement of Nova bone putty. Tissues were approximated using simple interrupted suture. Following the placement of a basal implant, the patient was given specific post-operative instructions to ensure proper healing and implant stability. Cold packs were advised to be applied intermittently for the first 24 hours to minimize swelling and discomfort. The patient was instructed to avoid biting directly onto the implant to prevent undue pressure and movement. Sutures were removed after seven days, and provisional acrylic restorations were delivered to restore function and esthetics during the healing phase. To prevent infections, the patient was prescribed Augmentin 625 mg (Amoxicillin 500 mg + Clavulanic Acid 125 mg) every 12 hours for five days. Pain and inflammation were managed with NSAID Diclofenac Potassium 50 mg every eight hours for five days. For oral hygiene maintenance, the patient was advised to rinse with Hexitol (Chlorhexidine 125 mg/100 ml, concentration 0.125%) to reduce bacterial load and promote healing. These measures were taken to ensure a smooth recovery and the long-term success of the implant, with regular follow-ups recommended monitoring progress.

## Discussion:

Basal Implantology signifies a transformative advancement in contemporary dentistry, particularly in scenarios where traditional implant placement is hindered by significant bone resorption. Unlike conventional implants that depend on the trabecular alveolar bone, basal implants utilize the dense cortical basal bone, offering enhanced stability and the potential for immediate functional loading. This approach is especially advantageous in the anterior region where esthetics, function, and soft-tissue preservation are of paramount importance. The occurrence of periodontal disease and subsequent tooth loss often results in ridge atrophy, posing significant challenges for conventional implant rehabilitation. However, basal implants bypass the need for extensive grafting procedures, thereby reducing surgical morbidity and total treatment duration. By anchoring into the cortical bone, which is less prone to resorption and infection, these implants provide enduring support even in compromised clinical conditions [6- 7]. Pathak *et al.* evidenced the advantages of basal implants in the anterior maxilla, particularly in cases with reduced buccolingual width, by demonstrating successful outcomes with immediate loading using flapless surgery. The authors stated that basal implants permit immediate temporization and are particularly beneficial where conventional implants fail due to anatomical limitations [8]. Garg *et al.* documented a 100% survival rate for basal implants placed with immediate loading over a three-year period, highlighting their clinical reliability even in patients with systemic conditions [9]. Likewise, Pathania *et al.* conducted a comparative study that reported a 99.6% survival rate for basal implants in fresh extraction sockets and a 99.0% rate in healed ridges, supporting the biological and clinical preference for immediate placement [10]. Furthermore, Ihde (2001) emphasized the success of basal osseointegrated implants in the rehabilitation of severely atrophied mandibles, suggesting that these implants are well-suited for challenging cases requiring stable anchorage and immediate function [11]. According to Wagner and Hartung (2021), the design of basal implants eliminates the implant-abutment microgap common in two-piece systems, thereby reducing the risk of bacterial colonization and peri-implantitis. Their polished surface and broad threads improve biomechanical stability and vascular support around the implant [12]. In the present case, the supplementary use of NovaBone Putty was instrumental in enhancing osseointegration and addressing local bone deficiencies. NovaBone, a synthetic calcium phosphate silicate material has osteoconductive property. As demonstrated in this case, it served as a scaffold for osteoblastic activity, minimizing micromotion and improving implant adaptation in regions with limited native bone [13].

The surgical approach was meticulously designed to be minimally invasive while maximizing preservation of the alveolar ridge. Atraumatic extraction of tooth 22 preserved the integrity of the surrounding structures. Immediate implant placement not only reduced treatment time but also prevented post-extraction bone collapse. The use of mid-crestal and pyramidal mucoperiosteal flaps allowed for precise access and control while maintaining the surrounding soft tissue envelope. Recent literature has emphasized the clinical and esthetic efficacy of immediate implant placement, particularly in cases involving severe alveolar ridge resorption. Patel *et al.* conducted a prospective study on the use of basal implants in atrophied maxillary and mandibular jaws, reporting success rates and patient satisfaction, thereby validating basal implants as a practical solution in compromised anatomical situations [14]. Supporting this, Awadalkreem *et al.* assessed patient satisfaction following treatment with immediately loaded basal implants and found a marked improvement in quality of life and functional outcomes, highlighting the psychological and prosthodontic benefits of early loading protocols in edentulous patients [15]. From an esthetic standpoint, Chen and Buser performed a systematic review evaluating immediate and early implant placement in the anterior maxilla and concluded that although both approaches yield acceptable outcomes, immediate implants in thin biotypes or deficient facial bone walls are more susceptible to midfacial recession, thereby demanding meticulous planning. Complementing these findings, Belser *et al.* reviewed implant restorations in the anterior maxilla and stressed the significance of three-dimensional implant positioning, soft tissue volume, and contour for long-term esthetic success [16]. Collectively, these findings reinforce the present case's treatment protocol, where basal implants provided immediate mechanical anchorage in a resorbed ridge, and NovaBone Putty supported osseointegration in deficient bone zones. The approach not only restored esthetics and function with minimal morbidity but also aligned with emerging evidence on patient-centered and time-efficient implant protocols.

## Conclusion:

This case shows the clinical benefits of using basal implants alongside NovaBone Putty for immediate rehabilitation in cases of ridge resorption. The method effectively reduces surgical morbidity and eliminates the need for complex augmentation procedures. It enables rapid restoration of both function and esthetics through a minimally invasive approach. Successful outcomes in challenging lower anterior cases highlight the predictable and effective nature of Basal Implantology. However, additional randomized controlled trials are essential to establish standardized protocols and assess long-term results.

## Source(s) of support:

Nil
